# Supplementary material for: A sense of belonging: The role of higher education in retaining quality STEM teachers
Source: PLoS One. 2022 Aug 17;17(8):e0272552. doi: 10.1371/journal.pone.0272552 (PMC9385046; doi:10.1371/journal.pone.0272552)
Supplement: Attachment — Submitted filename: Response to Reviewers (Balgopal-Weinberg et al-PLOS).docx [file pone.0272552.s001.docx]

Department of Biology Campus Delivery 1878

Fort Collins, Colorado 80523-1878 Telephone: (970) 491-7011

FAX: (970) 491-0649

[www.biology.colostate.edu](http://www.biology.colostate.edu/)

November 9, 2021

Dear Editorial Board,

We are resubmitting our research article, PONE-D-21-18404, “**A Sense of Belonging: The Role of Higher Education in Retaining Quality STEM Teachers**” for consideration in *PLOS One*.

Thank you for the opportunity to address reviewer comments. We carefully considered each comment as we substantially revised our manuscript. We believe that the manuscript is much clearer and will contribute to our understanding of how the pandemic has affected high-quality STEM teachers. Here, we highlight the types of support that professional and pre-service STEM teachers reported as shaping their decisions to remain or leave the teaching profession. Considering the national STEM teacher shortage, our study is important and timely.

Please see the following table that describes how we addressed each reviewer comment.

Thank you for your reconsideration of this manuscript.

With kind regards,

Meena M. Balgopal, Professor, Colorado State University

Andrea E. Weinberg, Assistant Professor, Arizona State University

| **Reviewer Comment** | **Response** |
| --- | --- |
| The manuscript, though important, needs to be rewritten and to strictly follow the comments made by the reviewers. | Thank you for the opportunity to revise our paper, which was initially framed as a data-supported commentary in which we made bold claims. However, in keeping with the research report format of PLOS One, we have used the feedback from the reviewers to revise how we interpreted and framed our results. |
| **Reviewer #1**:  Even this is a well-written article, the descriptive analysis used is not rigorous enough on exploring the issue under studied. | We appreciate the concerns of the reviewer and have provided more details about our analysis, including the inferential analyses, demographic details, and explanations of our results. |
| **Reviewer #2**: 1. The findings are not directly tied to the data analysis and there seems to be some bias present in the findings. | We have revised how we report the results as well as the claims being made. We revised any claims that may be perceived as overstated or overtly biased. |
| 2. The paper says data were analyzed using SPSS, but does not specify what kind of statistical analysis was done. Although the data says qualitative data was collected and analyzed, no qualitative data was presented. | We clarified that content analysis was used to code qualitative (open response survey items) data. Subsequently, we transformed codes into numerical counts that could be statistically analyzed in SPSS. |
| 3. The results section was difficult to follow, it took several reads to understand what was being presented. I recommend explaining what the 1A, 1B etc. in the body of the paper. | We addressed this edit in a couple of ways. First, we clarified the titles and axes in the Figure (now, Figure 2). Second, we added explanatory text in the Results section to help readers interpret the Figure, including the relevance of Boxplots A, B, and C. |
| **Reviewer #3**: Line 46 - "as decision are made" is vague. By whom? | We specified that the decisions referred to are those being made by policymakers, often without input from educators. |
| Lines 82-88 - some information on how data were aggregated is needed here. What were emerging themes, if any? | We expanded the description of emergent themes as we added additional information about the methods, including data analysis. |
| Figure 1: y-axes should be labeled. | The y-axis labels were added in what is now Figure 2. We made other edits to this figure to further clarify the graphs. We appreciate the feedback that prompted us to review our figure. |
| Discussion: some mention of design limitations is needed (specifically geographic limitations). | We have included limitations in the discussion. |
| Otherwise, I think the authors are overgeneralizing their findings. | We revisited the manuscript, paying particular attention to the findings section, and reworded or relocated any statements that are not fully supported by the data.  For example, we moved interpretations that describe the importance or relevance of findings to the discussion section (two sentences)  We added seven citations to bolster our claims. |
| Ideas for future research would be a nice addition to the discussion. | Areas for future research were added in the discussion. |
| Some information on regional demographics would also be good to characterize the economic state of where the data were collected | Additional details about participant demographics were added, including gender, race/ethnicity, status as having dependent(s), STEM teaching discipline, years of teaching experience, school type (e.g., public).  Although our participants were recruited from Noyce programs at universities in the US West, they are now teaching in schools across 18 different states and nearly every participant is teaching in a different district. Instead of providing regional demographics, we offered this information in the manuscript as an explanation to help readers understand why we chose not to discuss demographics beyond those at the teacher level in this manuscript. Furthermore, we included a new figure (a map indicating the states from which we recruited participants, where participants were teaching, and where no participants were teaching). The addition of this graphical figure highlights that our participants were, indeed, spread out across a broader geographic area than their teacher education programs (Noyce programs). |
| **Reviewer #4**: In order to evaluate this manuscript fully the following information is required: 1. more detail in the methods, especially about data analysis | As stated above, we specified the overarching data analysis approach (content analysis) and named the analyses conducted (e.g., t-tests and ANOVA) |
| 2. more details about participant demographics, including age, gender, race/ethnicity, etc | Additional details about participant demographics were added, including gender, race/ethnicity, status as having dependent(s), STEM teaching discipline, years of teaching experience, school type (e.g., public). |
