# Supplementary material for: A sense of belonging: The role of higher education in retaining quality STEM teachers
Source: PLoS One. 2022 Aug 17;17(8):e0272552. doi: 10.1371/journal.pone.0272552 (PMC9385046; doi:10.1371/journal.pone.0272552)
Supplement: Attachment — Submitted filename: Response to Reviewer Matrix March2022.docx [file pone.0272552.s002.docx]

| **Reviewer Comment** | **Response** |
| --- | --- |
| **Editor** |  |
| The study is suggesting commitment to school and profession indicating either normative, affective, continuance commitment (Meyer and Allen, 1997) framework and scale. This manuscript is not explicit about the validated scale used to collect the data. | Thank you for your suggestion. Meyer and Allen’s (1997) work is focused on organization-level commitment. Participants in our study included teachers across 18 states who are teaching at over 150 schools (across nearly as many districts). Each of these contexts is dramatically different and cannot be considered a single organization. We are looking beyond the organization (i.e., school) - both in terms of teacher motivations and the sources of support. Thus, this framework is tangential to our study.  The participants we recruited are all recipients of federal funding and were selected for their professional commitment. However, the pandemic has been such an unprecedented challenge for teachers that our motivation to conduct the study was to identify which teachers intended to leave after their scholarship commitments were fulfilled and to identify what might explain these intentions. |
| You mentioned three surveys were administered - there is a need to report the validation procedure on the scale - any data collected from non-valid measures are not acceptable | We added clarifying language about how the open-responses were used as a measure of the reliability of the closed-responses. We also extended an explanation about how we ensured content validity. |
| The introduction is also mentioning resilience in addition to commitment, which is another variable with other standard scale being used | A concern about professional resilience was the motivation to conduct the study. Resilience is not a construct that we were testing. Rather, it is presented in the literature as the practical outcome or motivation for the study. We revised the manuscript to clarify this, and we now explain that the study was exploratory, and, for those reasons, we were not testing variables or measuring constructs. Rather, we aimed to identify variables that might explain how teachers were responding to teaching during the pandemic.  We have included three citations to support the need for understanding resilience in general, and especially as it relates to a global pandemic.   1. Trust, T., & Whalen, J. (2021). K-12 teachers’ experiences and challenges with using technology for emergency remote teaching during the COVID-19 pandemic. *Italian Journal of Educational Technology. doi:10.17471/2499-4324/1192* 2. Hartshorne, R., Baumgartner, E., Kaplan-Rakowski, R., Mouza, C. & Ferdig, R.E. (2020). Special Issue Editorial: Preservice and Inservice Professional Development During the COVID-19 Pandemic. *Journal of Technology and Teacher Education, 28*(2), 137-147. Waynesville, NC USA: Society for Information Technology & Teacher Education. Retrieved February 28, 2022 from <https://www.learntechlib.org/primary/p/216910/> 3. Crompton, H., Burke, D., Jordan, K., & Wilson, S. W. G. (2021). Learning with technology during emergencies: A systematic review of K-12 education. *British Journal of Educational Technology. 52,* 1554– 1575. <https://doi.org/10.1111/bjet.13114> |
| The objectives should suggest the methodology | We have added a follow up sentence after the objectives before the methods section that clarify what the methodology is.  We also included a citation in the opening of the Methods section:   1. Switzer, G. E., Wisniewski, S. R., Belle, S. H., Dew, M. A., & Schultz, R. (1999). Selecting, developing, and evaluating research instruments. *Social Psychiatry and Psychiatric Epidemiology, 34*(8), 399-409. <https://doi.org/10.1007/s001270050161> |
| Page 5 suggest that you used mixed methods - qualitative and quantitative but you need to report the scale - the validation procedure of the scale | We explained that we used crossover analyses and included a citation.   1. Onwuegbuzie, A. J., Bustamante, R. M., & Nelson, J. A. (2010). Mixed research as a tool for developing quantitative instruments. *Journal of Mixed Methods Research, 4*(1), 56-78. |
| I suggest to have your data done by professional statistician and a qualitative researcher in order to improve the presentation. For example there is no mention what specific problems in which you used t-test and ANOVA useful? What are the mean scores of the variables being compared here and not, for example, on novelty or perceived impact | Our team comprises three scientists who have expertise in both quantitative and qualitative data analysis. To address the editor’s suggestions, we have shared our manuscript with two colleagues - a biostatistician and a psychometrician. Their feedback informed our edits. |
| For Lab, Study and Registered Report Protocols: These article types are not expected to include results but may include pilot data. | We have added the description that our study is exploratory in nature, and that pilot data are presented. |
| **Reviewer 2:** |  |
| You state in your figures that Pre-pandemic STEM graduates entering the teaching profession are supported by higher education and STEM networks. You need citations to support this claim | We have added citations in the introduction and discussion to support the claim in the figure.   1. Apriceno, M., Levy, S. R., & London, B. (2020). Mentorship during college transition predicts academic self-efficacy and sense of belonging among STEM students. *Journal of College Student Development, 61*(5), 643-648. 2. Master, A., & Meltzoff, A. N. (2020). Cultural stereotypes and sense of belonging contribute to gender gaps in STEM. *International Journal of Gender, Science and Technology, 12*(1), 152-198. 3. Skaalvik, E. M., & Skaalvik, S. (2011). Teacher job satisfaction and motivation to leave the teaching profession: Relations with school context, feeling of belonging, and emotional exhaustion. *Teaching and teacher education, 27*(6), 1029-1038. 4. Bastian, K. C., & Marks, J. T. (2017). Connecting teacher preparation to teacher induction: Outcomes for beginning teachers in a university-based support program in low-performing schools. *American Educational Research Journal, 54*(2), 360-394. 5. Basham, J. D., Israel, M., & Maynard, K. (2010). An ecological model of STEM education: Operationalizing STEM for all. *Journal of Special Education Technology*, *25*(3), 9-19. 6. Knowlton, S., Fogleman, J., Reichsman, F., & de Oliveira, G. (2015). Higher education faculty collaboration with K-12 teachers as a professional development experience for faculty. *Journal of College Science Teaching*, *44*(4), 46-53. 7. Richmond, G., Dershimer, R. C., Ferreira, M., Maylone, N., Kubitskey, B., & Meriweather, A. (2017). Developing and sustaining an educative mentoring model of STEM teacher professional development through collaborative partnership. *Mentoring & Tutoring: Partnership in Learning*, *25*(1), 5-26. |
| Figure 1A rather than saying "some teachers" include the N or percentage. | We have added percentages to the figure to reflect the text in the manuscript. |
| Figure 1B says professional STEM teachers were less likely than preservice to remain, but your survey asked about intentions, so it doesn't seem you have the data to support this claim. | We edited the figure to align with the manuscript text and reflect that the data were based on teacher intentions by including, *Professional STEM teachers were less likely than preservice teachers to anticipate remaining in STEM Education.*  *(Fig. 1B)*. |
| In the figure it says "A sense of isolation or belonging could impact teacher's..." this seems like opinion rather than data from your study. | We agree that this statement is conjecture and has been extrapolated from our data rather than based on the data. To address this, we had included tentative language and an inclusion of *Supported by Data* and *Extrapolated from Data*. However, to clarify the intent of including these statements, we have added *Extrapolated from data and warrants further study.* We have also included a citation in the manuscript to provide a literature-based rationale for this statement:   1. Borman, G. D., & Dowling, N. M. (2008). Teacher attrition and retention: A meta-analytic and narrative review of the research. *Review of Educational Research, 78*(3), 367-409. <https://doi.org/10.3102%2F0034654308321455> 2. Buchanan, John; Prescott, Anne; Schuck, Sandra; Aubusson, Peter; Burke, Paul; and Louviere, Jordan (2013) "Teacher Retention and Attrition: Views of Early Career Teachers," Australian Journal of Teacher Education: Vol. 38: Iss. 3, Article 8. |
| The N for teachers of color is really small, so I recommend not reporting findings on those statistics. (2 comments) | Although the numbers are small, they are still statistically relevant and practically significant, demonstrating power for any future studies with a larger sample size. There is a pressing need to understand how to recruit and support teachers of color — particularly in STEM disciplines, where attempts to broaden participation in STEM professions have been making minimal progress for decades. Representation (i.e., children seeing teachers who look like themselves) is important in K-12 schools, as this is when students’ STEM commitments are developed and solidified. |
| I don't see how the data supports the statement:  "Ultimately, many of today’s PK-12 students are future STEM professionals.  Because STEM teachers in American public schools are required to demonstrate their  content expertise by earning a degree in their licensure area, it is likely that many of  our undergraduate STEM students are future teachers." | We thank the reviewer for asking us to clarify our discussion. We removed the first sentence and revised the following to be more accurate.  *Because STEM teachers in American public schools are required to demonstrate their content expertise by earning a degree in their licensure area, future teachers are enrolled in our undergraduate STEM courses. it is likely that many of our undergraduate STEM students are future teachers*. |
| I don't see how the data supports the statement: "Our findings indicate that the  importance of how connected STEM teachers feel to their professional communities,  including university professors, may predict their intentions to remain in the  profession." Additionally, "may predict" does not inform any actionable interventions. | We clarified this claim to align with our results:  *Respondents in our study indicated that their feelings of being connected to their professional communities, including university professors, affected their intentions to remain in the profession.* |
| "Some teachers’ decisions to remain in teaching are influenced by their desire for both financial and professional stability, highlighting the need to pay STEM teachers salaries commensurate with other STEM  professions." this finding was only for TOC correct? | Yes, the finding was only for TOCs. We have decided to keep these results, but we clarified that although the results were only marginally significant, they were practically significant and warrant further exploration as others have argued regarding marginal p-values with small sample size.  We have added citations to support this decision:   1. Wong, P. M., & Leung, S. O. (2010). The development of theory of mind ability in autistic children. *Psychologia, 53*(3), 151-162. 2. Morgan, G. A., Gliner, J. A., & Harmon, R. J. (2006). *Understanding and evaluating research in applied and clinical settings*. Psychology Press. |
| **Reviewer 3:** |  |
| No comments |  |
| **Reviewer 4:** |  |
| No comments |  |
